# Mapping lung function in late-onset Pompe disease using label-free functional MRI

**DOI:** 10.1016/j.isci.2026.115136

**Published:** 2026-02-25

**Authors:** Lina Tan, Alexandra L. Wagner, Rafael Heiss, Nadine Bayerl, Jana Zschüntzsch, Adrian P. Regensburger, Frauke Alves, Matthias Türk, Sandy Schmidt, Robert Grimm, Matthias Vorgerd, Lara Schlaffke, Hannah Vogt-Wolz, Merle Claßen, Benjamin Stoecklein, Adrian Buehler, Joachim Woelfle, Michael Uder, Andreas Hahn, Alexander Mensch, Martin Winterholler, Regina Trollmann, Roman Raming, Ferdinand Knieling

**Affiliations:** 1 Department of Medicine 3 – Rheumatology and Immunology, University Hospital Erlangen, Friedrich-Alexander-Universität (FAU) Erlangen-Nürnberg, Erlangen 91054, Germany; 2 Department of Pediatrics and Adolescent Medicine, University Hospital Erlangen, Friedrich-Alexander-Universität (FAU) Erlangen-Nürnberg, Erlangen 91054, Germany; 3 Translational Pediatrics, Department of Pediatrics and Adolescent Medicine, University Hospital Erlangen, Friedrich-Alexander-Universität (FAU) Erlangen-Nürnberg, Erlangen 91054, Germany; 4 Derpartment of Pediatric Neurology, Center for Chronically Sick Children, Charité Berlin, Berlin 13353, Germany; 5 Institute of Radiology, University Hospital Erlangen, Friedrich-Alexander-Universität (FAU) Erlangen-Nürnberg, Erlangen 91054, Germany; 6 Neuromuscular Disease Research, Department for Neurology, University Medical Center Göttingen, Göttingen 37075, Germany; 7 Translational Molecular Imaging, Max-Planck Institute for Multidisciplinary Sciences, City Campus, Göttingen 37075, Germany; 8 Translational Molecular Imaging, Clinic for Haematology and Medical Oncology, Department for Clinical and Interventional Radiology, University Medical Center, Göttingen 37075, Germany; 9 Department of Neurology, University Hospital Erlangen:Friedrich-Alexander-Universität (FAU) Erlangen-Nürnberg, Erlangen 91054, Germany; 10 Centre for Rare Diseases Erlangen (ZSEER), University Hospital Erlangen, Friedrich-Alexander-Universität Erlangen-Nürnberg (FAU), Erlangen 91054, Germany; 11 Research and Clinical Translation, Magnetic Resonance, Siemens Healthineers AG, Erlangen 91042, Germany; 12 Department of Neurology, BG-University Hospital Bergmannsheil, Ruhr-University Bochum, Bochum 44789, Germany; 13 Heimer Institute for Muscle Research, BG-University Hospital Bergmannsheil, Bochum 44789, Germany; 14 Department of Child Neurology, University of Giessen, Giessen 35385, Germany; 15 Department of Neurology, University Medicine Halle, Halle (Saale) 06120, Germany; 16 Sana Krankenhaus Rummelsberg, Nürnberg/Schwarzenbruck 90489, Germany; 17 Center for Social Pediatrics, University Hospital Erlangen, Friedrich-Alexander-Universität (FAU) Erlangen-Nürnberg, Erlangen 91054, Germany

**Keywords:** health sciences, neuroscience

## Abstract

Pompe disease is a life-limiting metabolic myopathy characterized by proximal muscle weakness. In late-onset Pompe disease (LOPD), respiratory failure due to respiratory muscle weakness is the leading cause of death. Monitoring relies on spirometry, which is often not feasible in pediatric or severely affected patients, highlighting the need for effort-independent assessments. Phase-resolved functional lung (PREFUL) MRI enables non-invasive, label-free evaluation of lung function during free breathing without active cooperation. In this prospective pilot study, ten LOPD patients and ten age- and sex-matched healthy controls underwent 0.55 T PREFUL MRI. The method proved feasible and detected ventilation-related functional impairments in LOPD patients. Moreover, differences were observed depending on non-invasive ventilation (NIV) status. These findings align with the pathophysiology of respiratory muscle involvement in LOPD. PREFUL MRI may serve as a non-invasive imaging biomarker for respiratory dysfunction and overall disease burden in neuromuscular diseases.

## Highlights

• PREFUL MRI provides functional lung imaging and supports disease monitoring
• PREFUL MRI detects ventilation defects that reflect LOPD disease severity
• Respiratory support status influences ventilation defects
• PREFUL MRI is fast, well tolerated, and feasible in clinical routine

## Graphical abstract

**Figure:**
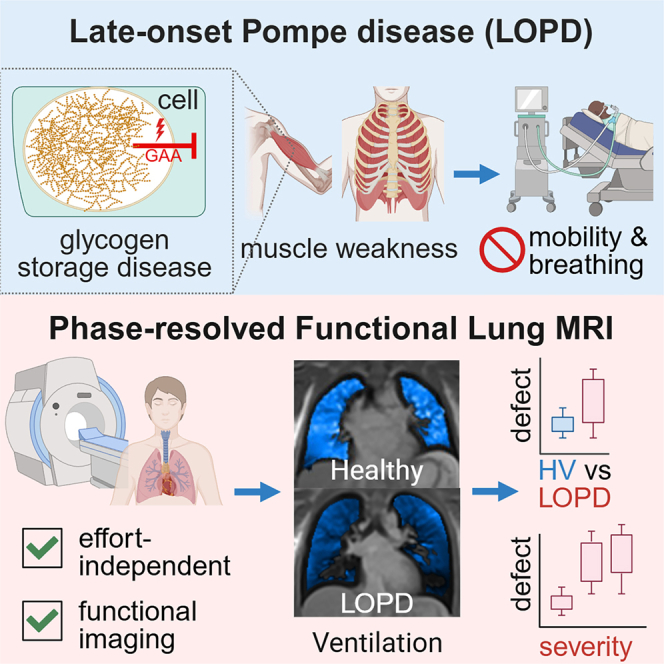

## Introduction

Pompe disease is a life-limiting, metabolic myopathy characterized by proximal limb-girdle muscle weakness and progressive respiratory insufficiency.^1^ It is caused by an autosomal recessive mutation in the acid alpha-glucosidase (GAA) gene, resulting in reduced GAA enzyme activity, impaired lysosomal glycogen degradation^2^ and subsequent irreversible cell damage.^1^^,^^2^^,^^3^ Residual GAA activity correlates inversely with disease severity,^4^ allowing classification into two major forms: infantile-onset (IOPD) with no and late-onset Pompe disease (LOPD) with reduced residual GAA activity. In more than 30% of LOPD cases, respiratory muscle weakness is the initial clinical manifestation.^5^^,^^6^ A characteristic feature of LOPD is early involvement of the diaphragm,^7^ resulting in respiratory insufficiency that is more pronounced in the supine position and manifests as sleep-disordered breathing and nocturnal hypoventilation.^8^ While disease progresses, respiratory failure commonly extends into the daytime, even while upright vital capacity (VC) may still remain normal.^1^ In this context, the use of ventilators for breathing assistance is well established, as respiratory failure substantially reduces quality of life and remains the leading cause of death in LOPD.^5^^,^^9^^,^^10^

Since 2006, causal treatment with enzyme replacement therapy (ERT) is available and significantly reduces mortality and improves functional status.^11^^,^^12^^,^^13^ Respiratory function often stabilizes under treatment while untreated individuals typically experience progressive decline in forced VC (FVC) and an increase in ventilatory support.^12^ In this regard, continuous evaluation of respiratory involvement, indicative of disease progression and treatment response is essential.^14^^,^^15^ In clinical practice, respiratory function is routinely monitored using screening questionnaires, manometry, and spirometry.^1^^,^^8^^,^^16^ During positional spirometry, diaphragmatic involvement can be inferred from a reduction in forced VC (FVC) in the supine position compared with the upright position. However, as spirometry is most often performed using stationary equipment in the seated position, diaphragmatic weakness is frequently underestimated. Moreover, conventional manometry and spirometry have several inherent limitations: they lack regional specificity, rely on active patient cooperation, and require technically reliable test execution. As a result, its feasibility is limited in pediatric patients, ventilated individuals, and those unable to sit.^11^

In contrast, magnetic resonance imaging (MRI) offers the advantage of directly assessing structural muscle involvement, with previous studies predicting respiratory insufficiency based on fatty muscle degeneration.^15^^,^^17^^,^^18^ Moreover, dynamic MRI techniques may capture thoracic and diaphragmatic motion.^19^^,^^20^^,^^21^ In LOPD, thoracic wall motion often remains preserved, while diaphragmatic motion and curvature are reduced during inspiration.^18^^,^^22^ These findings suggest relative sparing of the intercostal muscles.^18^ In addition, there is evidence that diaphragm dysfunction may be compensated by increased activity of the chest wall musculature.^7^ Therefore, dynamic MRI parameters appear to be more sensitive than conventional pulmonary function tests; however, they typically rely on breath-hold sequences, making active patient cooperation essential. This underscores an unmet clinical need for effort-independent methods capable of regionally assessing respiratory insufficiency in patients with Pompe disease across all ages and stages of disease severity.

Phase-resolved functional lung (PREFUL) MRI may provide a promising technology to address this gap. Based on conventional proton MRI, it is a further development of Fourier decomposition MRI. PREFUL MRI enables dynamic lung imaging under free-breathing by measuring signal intensity changes in each voxel over time.^23^ This signal intensity change is a superposition of (1) changes in proton density due to compression of the lung parenchyma over the respiratory cycle and (2) changes in magnetization caused by blood in-flow during the cardiac cycle. PREFUL separates these components into maps of ventilation and perfusion, and, based on an automatic deep learning-based segmentation of the lungs and pre-defined thresholds, allows quantification of regional defects in ventilation and/or perfusion. This approach allows effort-independent characterization of lung function, applicable across all PD patient populations.^23^^,^^24^ PREFUL MRI has been validated in multiple studies^25^^,^^26^ and has demonstrated high repeatability.^27^

In this study, we employed low-field (0.55T) PREFUL MRI to assess feasibility and detect functional alterations in patients with LOPD compared with age- and gender-matched healthy volunteers.

## Results

### Participants characteristics

**Figure 1:**
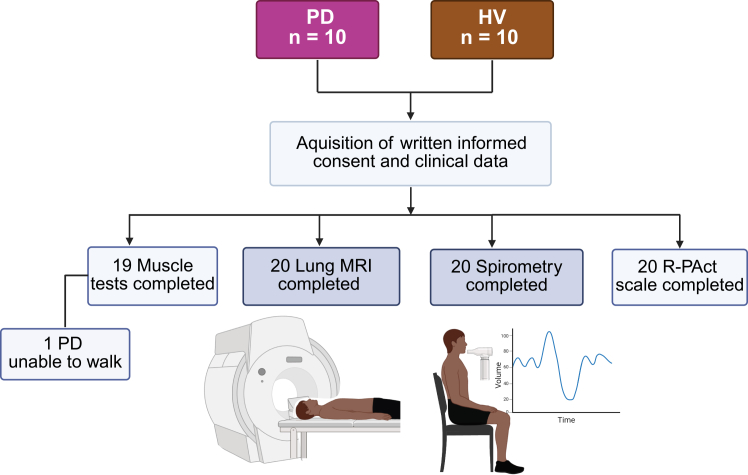
Study flow chart Flowchart diagram of the study. PD, Pompe disease; HV, healthy volunteers; MRI, magnet resonance imaging; R-PAct, Rasch-built Pompe-specific activity score. The figure was created using BioRender.com released under a Creative Commons Attribution-NonCommercial-NoDerivs 4.0 International license.

**Figure 2:**
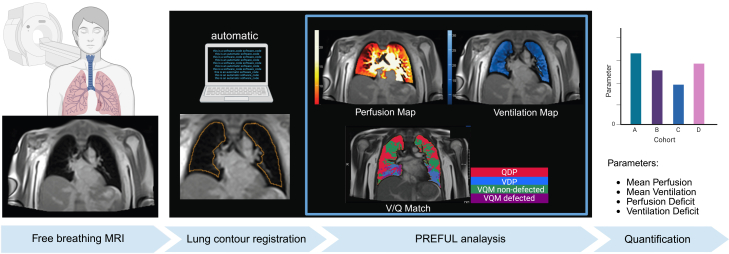
Imaging principle and data analysis Schematic representation of imaging approach. Free breathing phase-resolved functional lung (PREFUL) low-field strength magnetic resonance imaging (MRI) at 0.55 T was conducted. Midexpiration position of the lung was automatically registered. Lung contours were provided automatically by the MR lung 2.2 software (MR Lung version 2.0, Siemens Healthineers). No manual adjustments were performed for the study cohort. Parameters were calculated voxel-wise by using dedicated software. The figure was created using BioRender.com released under a Creative Commons Attribution-NonCommercial-NoDerivs 4.0 International license.

A total of *n* = 10 LOPD patients and *n* = 10 sex- and age-matched healthy volunteers (HV) were recruited at the Department of Children and Adolescent Medicine, Erlangen between May, 2022 and March, 2023 (Figure 1 and Table 1). All participants underwent PREFUL MRI (Figure 2) and functional testings. All data are presented as mean ± standard error of the mean (SEM). The mean age of LOPD patients was 40.6 ± 4.0 years compared to 41.2 ± 4.7 years in HV group. 50% of LOPD and HV were female. The mean body mass index (BMI) was 21.6 ± 1.3 in patients with LOPD and 23.6 ± 0.7 in HV. In the LOPD group *n* = 8 (80%) patients received ERT with and *n* = 2 (20%) received no ERT. *n* = 4 (40%) patients required nocturnal ventilation support.

**Table 1:** Demographic and clinical characteristics of the study participants

	Healthy volunteers *n* = 10	Pompe disease *n* = 10
Female (%)	5 (50)	5 (50)
Male (%)	5 (50)	5 (50)
Age, y	41.2 ± 4.7	40.6 ± 4.0
Weight, kg	73.9 ± 4.0	65.0 ± 5.8
Height, cm	176.2 ± 0.0	172.3 ± 0.0
BMI, kg/m^2^	23.6 ± 0.7	21.6 ± 1.3
Ambulatory (%)	10 (100)	9 (90)
ERT (%)	0 (0)	8 (80)
Nocturnal ventilation support	0 (0)	4 (40)
Lung function		
VC (%)	105.7 ± 3.4	63.3 ± 7.4
FVC (%)	104.9 ± 3.4	62.6 ± 7.4
FEV1 (%)	100.8 ± 3.7 3,738	68.2 ± 7.8
PEF (%)	111.4 ± 5.1	79.2 ± 8.1
R-PAct Scale	36.0 ± 0.0	29.1 ± 3.4
Functional testing		
QMFT	64 ± 0.0	46.7 ± 5.4
6MWT, m	664.7 ± 42.5	567.0 ± 31.9
TUAG, s	1.8 ± 0.2	3.7 ± 1.1
MRC		
UB proximal, medium, distal	5.0 (±0) – 5.0 (±0) – 5.0 (±0)	4.3 (±0.05) – 4.5 (±0.03) – 4.7 (±0.01)
LB proximal, medium, distal	5.0 (±0) – 5.0 (±0) – 5.0 (±0)	3.9 (±0.02) – 4.1 (±0.03) – 4.5 (±0.02)
PREFUL MRI		
Mean Perfusion (%)	10.7 ± 1.0	10.0 ± 0.9
Mean Ventilation (%)	16.6 ± 2.4	10.6 ± 0.8
QDP (%)	4.0 ± 2.0	5.2 ± 1.9
VDP (%)	11.0 ± 1.9	17.0 ± 2.8
VQM (Defect) (%)	0.2 ± 0.1	0.6 ± 0.2
VQM (Non-Defect) (%)	85.2 ± 2.8	78.4 ± 3.5

Values are mean ± standard error of mean (SEM). The reported MRC value represents the mean of multiple proximal, medium, and distal muscle groups of the upper and lower body. BMI, body mass index; ERT, enzyme replacement therapy; VC, vital capacity; FVC, functional vital capacity; FEV1, forced expiratory volume; PEF, peak expiratory flow; R-PAct, Rasch-built Pompe-specific activity score; QMFT, quick motor function test; 6-MWT, 6-minute walking test; TUAG, timed up-and-go test; MRC, medical research council score; UB, upper body; LB, lower body; QDP, perfusion defect percentage; VDP, ventilation defect percentage; VQM, ventilation perfusion match.

### Functional and pulmonary status is impaired in LOPD

Next, we aimed to evaluate the functional status of all *n* = 20 participants by completing the Rasch-built Pompe-specific activity scale (R-Pact). The LOPD group presented significantly lower scores, indicating substantial disease-related impairment in performing everyday tasks compared to HV (LOPD vs. HV: 29.1 ± 3.4 vs. 60.0 ± 0.0, *p* = 0.0611). Then, we evaluated muscle strength and motor function with general assessments such as timed up and go test (TUAG), 6-minute-walk test (6-MWT), and muscle strength testing using the Medical Research Council (MRC) scale. Due to one case of paralysis in the LOPD group, the TUAG was performed by *n* = 9 LOPD patients and *n* = 10 HV and the 6-MWT by *n* = 9 LOPD patients and *n* = 9 HV. In these assessments, LOPD patients achieved lower scores, consistent with reduced muscle strength and motor function (Table 1). In addition, we assessed the Quick Motor Function Test (QMFT), a PD-specific measure of muscle function, in all participants. Patients with LOPD showed significantly lower scores compared to HV (LOPD vs. HV: 46.7 ± 5.4 vs. 64.0 ± 0.0, *p* = 0.0047) No adverse events were recorded during the study.

We then aimed to assess lung function using spirometry. All participants (*n* = 20) were investigated in seated position. We found that all spirometric indices were significantly lower in patients with LOPD compared to HV. VC was 63.3% ± 7.4 and functional VC (FVC) 62.6% ± 7.4 in LOPD compared to VC of 105.7% ± 3.4 and FVC of 104.9% ± 3.4 in HV. Both differences were statistically significant with *p* < 0.0001. In LOPD, the forced expiratory volume in 1 s (FEV1) was 68.2% ± 7.8 and peak expiratory flow (PEF) was 79.2% ± 8.1. In HV, FEV1 was 100.8% ± 5.1, *p* < 0.0001 and PEF 111.4% ± 5.1, *p* = 0.0035.

## PREFUL MRI is feasible and resolves lung function in LOPD

**Figure 3:**
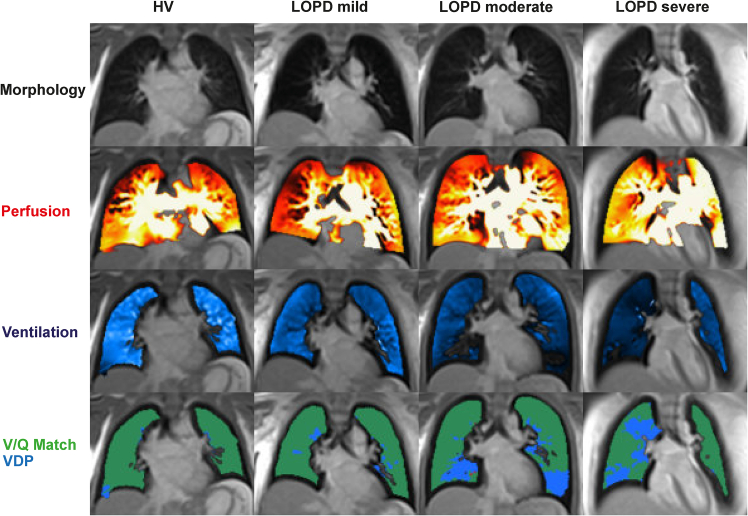
Representative PREFUL MRI images based on disease severity Free-breathing phase-resolved functional lung (PREFUL) low-field-strength magnetic resonance imaging (MRI) at 0.55 T, with calculated parameters in the axial plane after automatic registration to a mid-expiration position and lung parenchyma segmentation. From left to right, representative MRI lung morphology of a healthy volunteer (HV) and a LOPD patient of increasing disease severity according to their vital capacity (VC). We classified patients with a normal VC of 100%–88% as LOPD mild, patients with a VC of 87%–58% as LOPD moderate, and patients with a VC of 57%–0% as LOPD severe. From top to bottom, representative color-coded images from functional lung MRI show perfusion, ventilation, ventilation defects (blue), and ventilation-perfusion (V/Q) match (green). VDP, ventilation defect percentage.

**Figure 4:**
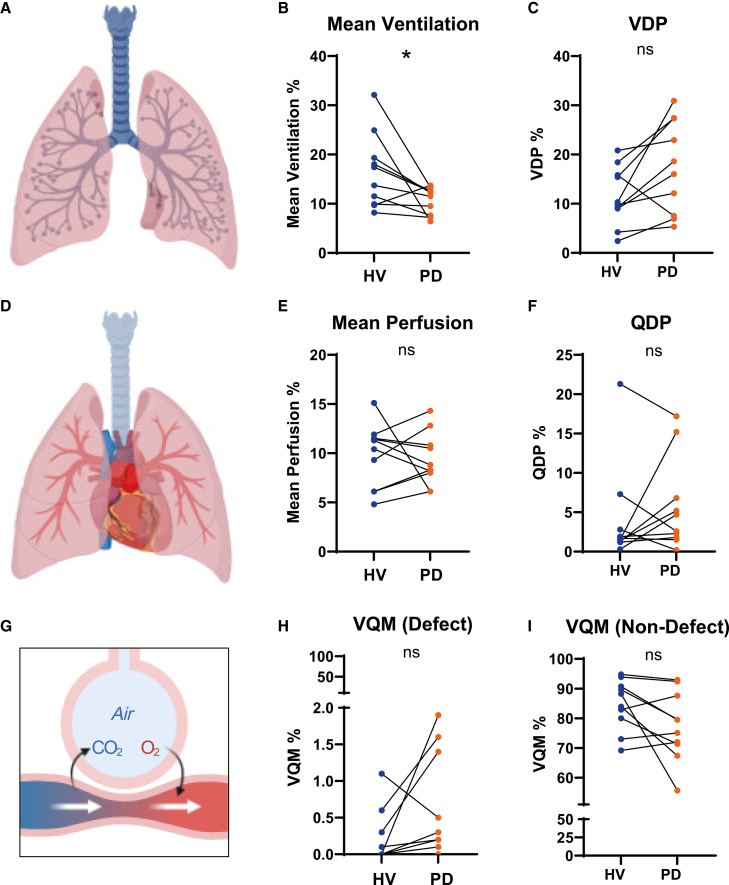
Statistical analysis of PREFUL MRI parameters (A, D, and G) From top to bottom: Illustration of measured low-field strength PREFUL MRI parameters: ventilation, perfusion, and ventilation-perfusion match of the lung. Comparison of PREFUL values for *n* = 10 healthy volunteers (HV) and *n* = 10 patients with LOPD for: (B) mean ventilation, (C) ventilation defect percentage (VDP), (E) mean perfusion, (F) perfusion defect percentage, (H) ventilation-perfusion match VQM (defect), (I) ventilation-perfusion match VQM (non-defect). Each orange-filled circle represents a single data point or measurement for a PD patient, connected to the corresponding age- and sex-matched healthy volunteer (blue-filled circle). Statistical analyses were performed using an unpaired *t* test or Mann-Whitney U test, depending on data distribution. ∗ = *p* < 0.05, ns = not significant.

Next, all *n* = 20 participants underwent low-field MRI to assess lung morphology, perfusion, and ventilation. Morphological assessment of the lung parenchyma was provided by a board-certified radiologist with 8 years of experience (R.H.). No pathological pulmonary findings were detected by MRI in either the HV or LOPD group. The principle of PREFUL imaging is illustrated in Figure 2. An overview of PREFUL measures is provided in Table 2. Representative functional MRI and morphological sequences for HV and LOPD of increasing respiratory involvement are shown in Figure 3. Representative functional low-field-strength MRI videos reveal differences in ventilation and perfusion in HV and LOPD (Videos S1 and S2). Quantitative analysis revealed significantly lower mean ventilation parameters in LOPD patients compared to their healthy matches (LOPD vs. HV: 10.65% ± 0.86% vs. 16.47% ± 2.4%, *p* = 0.0346; Figure 4B). To further compare the functional lung defects, we calculated the percentage in ventilation defects (VDP). In LOPD larger ventilation defects were observed compared to HV (LOPD vs. HV: 17.49% ± 3.0% vs. 11.53% ± 1.9%, *p* = 0.1078; Figure 4C). Mean perfusion values were similar in LOPD vs. HV: 9.39% ± 0.9 vs. 9.79% ± 1.0%, *p* = 0.7667; Figure 4E), whereas perfusion defects (QDP) were larger in LOPD compared to HV (LOPD vs. HV: 5.75% ± 1.9% vs. 4.07% ± 2.0%, *p* = 0.2250, Figure 4F). Next, we calculated the percentage of match between ventilated and perfused lung areas (VQM). We observed larger VQM defects in LOPD compared to HV, (LOPD vs. HV: 0.62 ± 0.2 vs. 0.21 ± 0.1, *p* = 0.0894; Figure 4H), though the results were not statistically significant. In contrast, non-defected ventilation and perfusion matches (VQM non-defect) did not show significant differences between groups (*p* > 0.05; Figure 4I). Thus, our results suggest that this technique is capable to visualize and quantify ventilation-related functional impairment, while isolated perfusion defects were not depicted. This finding aligns with the pathophysiology of the disease, which is primarily characterized by respiratory insufficiency due to muscular weakness rather than pulmonary vascular impairment.

**Table 2:** Overview and definition of PREFUL MRI measures

V	Ventilation
Q	Perfusion
VDP	Ventilation defect percentage
QDP	Perfusion defect percentage
VQM	Ventilation-perfusion match
VQM defect	Defect based on VQM
VQM non-defect	Non-defect based on VQM

Video S1. Representative lung ventilation patterns, related to Figure 3 Ventilation comparison of healthy individuals and mildly, moderately, and severely affected LOPD patients acquired during free breathing using phase-resolved functional lung (PREFUL) MRI at 0.55 T.

Video S2. Representative lung perfusion patterns, related to Figure 3 Perfusion comparison of healthy individuals and mildly, moderately, and severely affected LOPD patients acquired during free breathing using phase-resolved functional lung (PREFUL) MRI at 0.55 T.

### Ventilation defects reflect disease severity in LOPD

**Figure 5:**
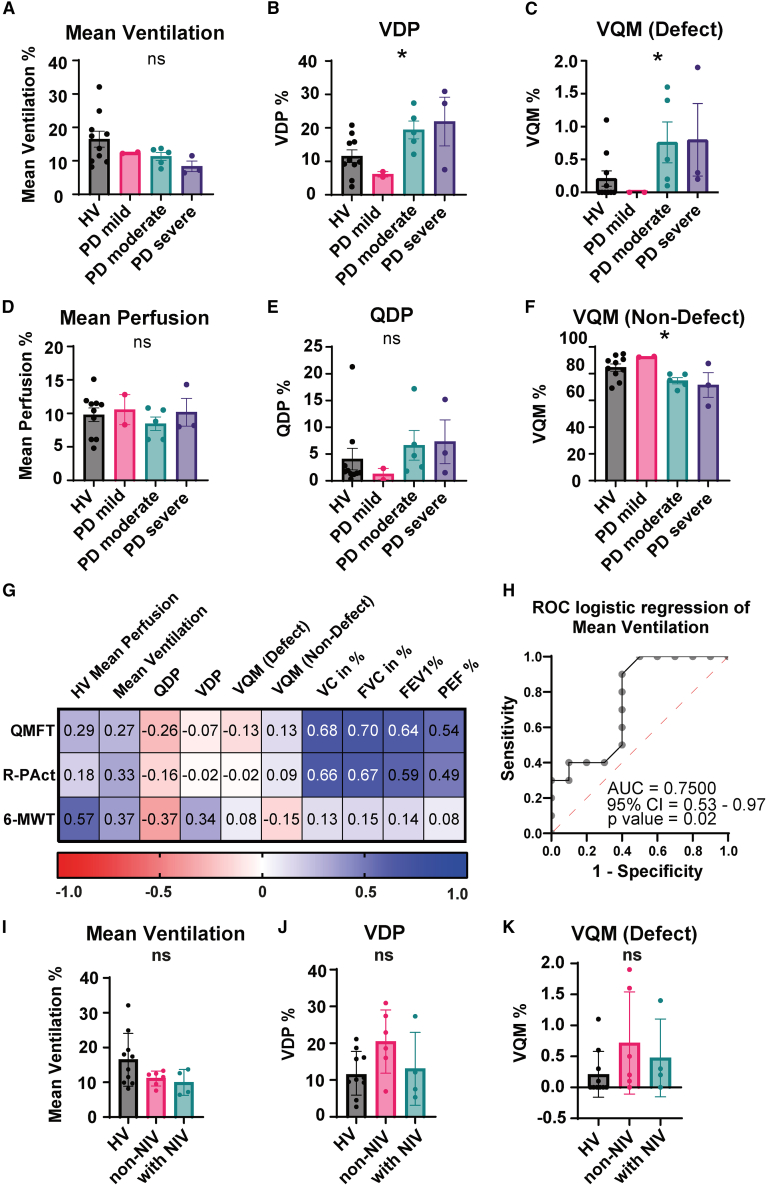
Statistical subanalysis of PREFUL MRI parameters *n* = 20 data points or measurements (*n* = 10 HV, *n* = 10 PD) were divided according to disease severity based on their vital capacity (VC): VC 100%–88% = PD mild (*n* = 2), VC 87%–58% = PD moderate (*n* = 5), VC 57%–0% = PD severe (*n* = 3). (A–F) Each bar displays the PREFUL MRI value for a whole group, with error bars indicating SEM. The color coding is as follows: black bar/filled circle = HV, pink bar/filled circle = PD mild, green bar/filled circle = PD moderate, violet bar/filled circle = PD severe. The parameters are: (A) mean ventilation, (B) ventilation defect percentage, (C) ventilation-perfusion match (defect), (D) mean perfusion (E) perfusion defect percentage (F) ventilation-perfusion match (non-defect), (G) correlation matrix for MRI and spirometric measures of the lung (mean perfusion, mean ventilation, perfusion defect percentage, ventilation defect percentage, ventilation-perfusion match (defect and non-defect), vital capacity (VC), functional vital capacity (FVC), forced expiratory volume in 1 s (FEV1), peak expiratory flow (PEF), correlated with reference clinical parameters of muscle: quick motor function test (QMFT), R-Pact scale, 6-min walk test (6-MWT). Correlations are indicated in the color range from highly negative (red) to low negative/positive (white) to highly positive (dark blue). (H) Logistic regression as ROC curve for mean ventilation. (I–K) *n* = 20 data points or measurements (*n* = 10 HV, *n* = 10 PD) were divided according to their need for nocturnal ventilation. Each bar displays the PREFUL MRI value for a whole proband group, with error bars indicating SEM. The color coding is: black bar/filled circle = HV, pink bar/filled circle = no nocturnal ventilation needed, green bar/filled circle = nocturnal ventilation needed. The parameters are: (I) mean ventilation, (J) ventilation defect percentage, (K) ventilation-perfusion match (defect). Group comparisons were analyzed by one-way ANOVA. Correlations are given by the Spearman correlation coefficient (rs), two-tailed test. *p* ≤ 0.05 was considered statistically significant. ∗ = *p* < 0.05, ns = not significant.

To further investigate the patient cohort, we stratified LOPD patients based on their VC. LOPD patients with normal VC of 100%–88% were classified as mildly affected (*n* = 2), those with a VC of 87%–58% moderately affected (*n* = 5), and those below 58% severely affected (*n* = 3). We observed that mean ventilation values declined with decreasing VC compared to HV (HV vs. LOPD mild vs. LOPD moderate vs. LOPD severe: 16.47 ± 2.4 vs. 12.40 ± 0.2 vs. 11.32 ± 1.2 vs. 8.4 ± 1.6, *p* = 0.18; Figure 5A). In contrast, VDP showed a statistically significant increase with disease severity, with the highest values found in the moderately and severely affected LOPD patients (LOPD moderate vs. LOPD severe: 19.40% ± 2.7% vs. 21.90% ± 7.3%), while mildly affected patients showed values comparable to HV (LOPD mild vs. HV: 6.10% ± 0.8% vs. 11.53% ± 1.9%, *p* = 0.04; Figure 5B). Similarly, VQM defect was significantly elevated in these two subgroups compared to mildly affected LOPD and HV (HV vs. LOPD mild vs. LOPD moderate vs. LOPD severe: 0.21 ± 0.1 vs. 0.0 ± 0.0 vs. 0.76 ± 0.3 vs. 0.8 ± 0.6, *p* = 0.02; Figure 5C). VQM defect values exhibited considerable inter-individual variability across the subgroups HV, LOPD moderate, and LOPD severe. Mean perfusion values and QDP were comparable across all subgroups and QDP showed considerable inter-individual variability (all *p* > 0.05; Figures 5D and 5E). A statistically significant decrease with disease severity was observed in non-defected VQM (HV vs. LOPD mild vs. LOPD moderate vs. LOPD severe: 84.62 ± 2.7 vs. 92.65 ± 0.3 vs. 74.74 ± 2.3 vs. 71.57 ± 9.2, *p* = 0.02; Figure 5F). We then correlated the PREFUL MRI parameters to functional tests, and found stronger correlation with the 6-MWT. In comparison, spirometric parameters demonstrated only weak 6-MWT correlation but the strongest correlations to Pompe-specific assessments QMFT and R-PAct (Table 1, Figure 5G). This may be explained by the physiological and methodological differences between these evaluations, suggesting that PREFUL MRI potentially reflects systemic disease burden rather than focusing solely on respiratory muscle dysfunction, while conventional spirometry reflects proximal muscle and respiratory weakness in seated position of LOPD patients. Among all PREFUL parameters, mean perfusion demonstrated the strongest correlation (Figure 5G).

### Ventilation impairment varies with respiratory support status in LOPD

Finally, we aimed to assess lung function in relation to the need for nocturnal non-invasive ventilation (NIV). Patients were stratified into those receiving NIV (“with NIV”, *n* = 4) and those not requiring ventilatory support (“non-NIV”, *n* = 6), and compared to HV. Mean ventilation was comparable among all subgroups (Figure 5I). Interestingly, the highest values for VDP and VQM defect were observed in the non-NIV group, exceeding those of both the with NIV group and HV (HV vs. LOPD non-NIV vs. LOPD with NIV: VDP 11.53% ± 1.9% vs. 20.45% ± 3.5% vs. 13.05% ± 5.0%, *p* = 0.09; Figure 5J; VQM defect 0.21 ± 0.1 vs. 0.72 ± 0.3 vs. 0.48 ± 0.3, *p* = 0.22; Figure 5K). These results indicate that non-nocturnal ventilated LOPD patients exhibit more pronounced ventilation-related impairments compared to both nocturnal ventilated patients and HV. However, these findings should be interpreted with caution and require further studies in larger, independent cohorts.

## Discussion

Our study demonstrates the potential and feasibility of PREFUL MRI as a patient-friendly, easily implementable and effort-independent imaging modality to assess lung function and respiratory involvement in PD patients. PREFUL MRI infers ventilation from motion-related signal changes during free breathing and has been performed in obstructive pulmonary diseases,^28^^,^^29^^,^^30^^,^^31^ cystic fibrosis,^32^^,^^33^^,^^34^^,^^35^ bronchiectasis,^36^ and COVID-19.^37^ The average examination time of PREFUL MRI was 1:30 min (Table S1) and was well tolerated by all probands, underscoring its suitability for all patients.

In our cohort, morphological assessment of the lung parenchyma revealed no pathologic findings, while functional analysis with PREFUL indicated ventilation-related impairments in LOPD. In contrast, perfusion parameters showed no significant abnormalities. These findings suggest that the observed defects are functional rather than morphological, consistent with the underlying etiology, pathophysiology, and clinical presentation of LOPD, as heart failure is uncommon.^38^ Notably, our results show larger ventilation defects and ventilation-perfusion mismatches in non-nocturnally ventilated patients compared to nocturnally ventilated patients. This may suggest that asymptomatic patients already experience ventilation-related impairments in supine position and could potentially benefit from earlier initiation of nocturnal ventilatory support.

Evaluation and optimizing therapy is standardized through the “triple-S” criteria and rely on longitudinal assessment of respiratory function,^14^ making long-term monitoring of respiratory involvement relevant. Cipaglucosidase alfa plus Miglustat (Pombiliti plus Opfolda, Amicus Therapeutics), and Avalglucosidase alfa (Nexviazyme, Sanofi) as new ERTs offer enhanced affinity, resulting in more efficient cellular uptake and prolonged FVC stabilization.^39^^,^^40^^,^^41^^,^^42^^,^^43^^,^^44^ Furthermore, gene therapies are emerging and have been successfully applied in patients with infantile-onset Pompe disease, resulting in significant functional improvement.^45^^,^^46^^,^^47^ Thus, comprehensive diagnostic approaches applicable to all patients, regardless of age and disease severity, are required.

As an effort-independent imaging method, PREFUL MRI has been applied to neonates, the youngest being three months old, and children without the need for sedation, demonstrating good feasibility.^37^^,^^48^ Its additional potential as a longitudinal biomarker has been shown in adolescents with cystic fibrosis, whose respiratory function under treatment was monitored over 24 months.^34^ These studies confirm the applicability of PREFUL MRI in pediatric patients. An effort-independent alternative is the electrical impedance tomography (EIT). Compared with MRI, EIT offers higher temporal resolution, enabling bedside assessment of pulmonary ventilation and perfusion in real time. This advantage makes it particularly useful in intensive care settings, where real-time treatment adjustments are particularly valuable.^49^ In neonates, EIT monitoring has been shown to reduce of ventilator-associated lung injury.^50^ However, the main limitations of EIT are the poor spatial resolution and the lack of standardized protocols, which currently hinder broader clinical implementation.

In contrast, PREFUL implementation requires only standard MRI hardware and a conventional GRE pulse sequence.^48^ PREFUL has shown to be repeatable and consistent across centers with a spatial overlap across centers of at least 91%, if images were acquired using the same field strength. Ventilation-related metrics differ when measured at different field-strengths^51^; therefore, multicenter studies require efforts toward standarization.^26^^,^^52^ In this study, all patients underwent 0.55 T MRI, which has advantages for morphologic lung imaging compared with 1.5 T and 3 T systems.^53^ PREFUL MRI has been validated against gadolinium-based,^54^ hyperpolarized^55^ and ^19^F MRI and demonstrated moderate to strong correlations and good spatial correspondence.^25^^,^^48^^,^^54^^,^^56^^,^^57^ While PREFUL-derived metrics correlated well with spirometry measures,^25^^,^^33^^,^^54^^,^^55^ this was not observed in our study. This discrepancy may be attributed to the LOPD-specific diaphragmatic involvement and differences in patient positioning during seated spirometry versus supine MRI. Interestingly, we observed distinct correlations regarding muscle testing: Pompe-specific measures of proximal muscle strength reflected more closely by spirometric indices, whereas the 6-MWT was correlated more closely, but not strongly, to PREFUL-derived metrics. This indicates that PREFUL-derived ventilation and perfusion parameters do not reflect muscle strength, but rather overall endurance and cardiorespiratory function.

### Limitations of the study

However, this feasibility study has limitations. As our findings indicate discrepancy between sitting spirometry and PREFUL-derived metrics, supine measurements should be included in future studies. For further validation, comparisons with perfusion/ventilation scintigraphy, or EIT could strengthen the confidence in PREFUL imaging for assessing LOPD patients. Another key limitation relates to the study cohort. Due to the rarity and variability of LOPD, the study included only a small and heterogeneous patient size. In addition, only adult patients were examined, which limits the generalizability of the findings to IOPD or pediatric/adolescent LOPD. Larger and more stratified cohorts are needed to confirm these results and standardize the methodology for broader clinical application. Nonetheless, this study provides a foundation for future research aimed at monitoring disease progression and therapeutic response. PREFUL-MRI could complement routine assessments, especially in vulnerable cohorts.

In conclusion, PREFUL MRI is a promising imaging modality for supporting assessment of respiratory involvement in LOPD. Its effort-independent nature makes it suitable for all PD patients, regardless of age or disease severity, and allows for longitudinal monitoring. In the future, PREFUL MRI could support therapeutic decision-making along with established standard tests. Furthermore, it presents low implementation barriers, both in terms of technical requirements and patient compliance, and can be easily integrated into clinical routine. Given its diagnostic and translational potential, PREFUL could also be extended to other neuromuscular diseases with respiratory involvement.

## Resource availability

### Lead contact

Further information and requests for resources can be directed to the lead contact, Ferdinand Knieling (ferdinand.knieling@uk-erlangen.de).

### Materials availability

This study did not generate new unique reagents.

### Data and code availability

This paper does not report original code. Due to participant confidentiality and privacy concerns regarding the participants, the data reported in this study cannot be deposited in a public repository. However, the data are available upon written request. Any further information necessary to reanalyze the data reported presented in this paper can be obtained from the lead contact upon request.

## Acknowledgments

The research was supported by a research Grant from Sanofi Aventis (SGZ201912542) for A.L.W., R.R., R.T., and F.K. Further support was provided by the 10.13039/501100000781European Research Council under the 10.13039/501100000780European Union Horizon H2020 program (ERC starting grant no. 101115742-IseeG) for F.K. and an Else Kröner Excellence Fellowship from the Else-Kröner Fresenius Stiftung for A.P.R. A.L.W. was supported by the Rahel Hirsch Program scholarship from the Charité University Hospital Berlin. The sponsors of the study had no influence on study design, data collection, and analysis or manuscript writing. The authors thank the Imaging Science Institute Erlangen for providing us with measurement time and technical support.

This study was supported by Sanofi Aventis.

## Author contributions

L.T., A.L.W., A.P.R., R.R., and F. K. designed, performed experiments, and clinical studies. S.S. performed clinical studies. R.H., N.B., J.Z., F.A., M.T., J.W., M.U., A.H., A.M., M.W., and R.T. provided essential support to the clinical study. R.G., M.V., and L.S. provided essential materials or technical expertise. L.T., R.R., A.L.W., and F.K. analyzed and interpreted the data. H.V., M.C., B.S., and A.B. provided support on data interpretation and illustration. F.K. conceived and supervised the project. L.T. and F.K. wrote the first draft of the manuscript. All authors edited and approved the final draft.

## Declaration of interests

A.L.W., A.P.R., and F.K. report travel supports from Sanofi Aventis, Germany. J.Z., A.P.R., M.T., and F.K. report lecture fees from Sanofi Genzyme. J.Z. reports travel support from Amicus Therapeutics and Sanofi Genzyme. J.Z. and M.T. report advisory board honoraria from Amicus Therapeutics and Sanofi Genzyme. A.M. reports speaking fees and advisory board honoraria from Hormosan Pharma and Sanofi-Aventis, and speaking fees from Amicus Therapeutics. R.G. is an employee of Siemens Healthineers AG. F.K. reports lecture fees from Siemens Healthcare GmbH.

## STAR★Methods

### Key resources table

**Table:** 

REAGENT or RESOURCE	SOURCE	IDENTIFIER
**Software and algorithms**		

MR Lung 2.2.0	Siemens Healthineers, Erlangen, Germany	–
MAGNETOM Free.Max 0.55T MRI	Siemens Healthineers, Erlangen, Germany	–
GraphPad Prism v10.2.0	GraphPad Software, La Jolla, CA, USA	–
BioRender	BioRender.com	–

### Experimental model and study participant details

*n* = 10 LOPD and age- and sex-matched *n* = 10 HV were recruited at the Pediatrics and Adolescent Hospital, Erlangen after having provided written and dated informed consent. Eligibility criteria included an age of over 18 years and for LOPD patients a confirmed genetic or enzymatic diagnosis. LOPD patients were included independent from current therapy. Exclusion criteria comprised pregnancy, and contraindications for MRI and for HV the absence of anamnestic or clinical signs of myopathy. The sex of participants was self-reported. The mean age ±SEM was 41.2 ± 4.7 years in HV compared to 40.6 ± 4.0 years in the LOPD patients’ cohort. In each group, 5 [50%] subjects were self-reported females. *n* = 8 LOPD patients were under ERT. *N* = 4 LOPD patients received nocturnal ventilation.

### Method details

#### Study design

This prospective, proof-of-concept study was conducted after receiving approval by the local ethics committee of the University Hospital Erlangen, Germany (no. 21–238_1B) and registered at clinicaltrials.gov (ID NCT05083806). The study was conducted in accordance with the Declaration of Helsinki during a single visit between May, 2022 and March, 2023. All participants underwent standard clinical assessment, spirometry and MRI imaging. Clinical assessment included the R-PAct, muscle strength test using the MRC scale and QMFT, TUAG and 6-MWT. To assess lung function, participants underwent spirometry in seated position. PREFUL-MRI was performed in supine position to evaluate ventilation and perfusion of the lungs.

Due to the pilot nature of the study, no sample size calculation was performed prior to enrollment. The primary outcome measure was the comparison of lung function, as detected by PREFUL-MRI, between PD patients and HV.

#### Clinical standard assessments

The R-PAct scale is a PD-specific and patient-reported questionnaire that comprises of 18 daily life activities items, ranging from “combing hair” to “running”. Each item is rated on a scale from (0) “unable to performs” to (1) “able to perform, but with difficulty” to (2) “able to perform without difficulty”. The R-PAct Scale is externally validated, reliable and demonstrated good discriminating ability.^58^ The QMFT is a PD-specific muscle function test in which participants complete 16 movement tasks (e.g., sitting up from a lying position, getting up from a chair). Each task is rated on a scale from 0 (no muscle contraction) to 4 (normal movement), with a maximum score of 64 points. The QMFT is sensitive to proximal muscle weakness and differences in disease severity. Muscle strength was evaluated bilaterally according to the MRC scale in proximal muscles of the upper and lower body. A total score was calculated by summing the scores for all 18 assessed muscle functions, rated from 0 (no muscle contraction) to 5 (normal strength), leading to a maximum of 180 points. The TUAG assessed the time taken to transition from a seated position to standing and completing a single step.^59^ The 6-MWT measures the distances covered in 6 min with walking aids allowed as needed.

#### Lung function

All subjects underwent conventional spirometry in seated position to quantify lung function. Parameters, such as VC, FVC, FEV1 and PEF, were assessed considering each person’s age, sex and weight. An average of all parameters was calculated out of a minimum of three measurements.

#### MRI protocol

All study participants received low-field MRI (0.55T MAGNETOM Free.Max, Siemens Healthineers) to visualize lung morphology and lung function.^25^^,^^27^^,^^35^^,^^37^^,^^51^ In all examinations a standard body coil was used for free-breathing lung imaging with a balanced steady-state free precession pulse sequence. The final parameters consisted of a two-dimensional central coronal section positioned at the midpoint of the lung hila: thickness of 15 mm, in-plane resolution of 1.7 × 1.7 mm, matrix size of 128 × 128 (interpolated to 256 × 256), bandwidth of 1149 Hz per pixel, flip angle of 80°, repetition time/echo time of 292.8/1.6 msec, parallel imaging acceleration factor of 2, no partial Fourier , 250 time points, temporal resolution of 300 msec, and an examination duration of 1 min and 30 s. For all MRI data and analyses, the evaluating radiologist (R.H., with 8 years of experience) was blinded to the clinical details. All study participants tolerated PREFUL MRI without any reported discomfort or adverse events.

#### Morphological and functional lung imaging

Sequences for morphological lung evaluation were acquired using a coronal and transversal turbo spin-echo sequence with periodically rotated overlapping parallel lines with enhanced reconstruction (BLADE) readout and respiratory gating. The coronal sequence was obtained using a short-inversion time inversion-recovery preparation with T2 weighting (repetition time/echo time, 2500/74 msec), 1.5 × 1.5 mm in-plane resolution, a 272 × 272 matrix, and a 6-mm section thickness. The transversal sequence was proton density–weighted (repetition time/echo time, 2000/33 msec) with 1.3 × 1.3 mm in-plane resolution, a 304 × 304 matrix, and a 6-mm section thickness.

For imaging of lung function, participants underwent free-breathing phase-resolved functional lung MRI (PREFUL). Various parameters were calculated voxel-wise. A detailed overview and explanation of all parameters used are provided in Table 2. The average PREFUL MRI scanning duration was 1:30 min. An overview of each participant’s PREFUL MRI scanning duration is provided in Table S1. The examination was tolerated by *n* = 20 probands.

#### Data analysis of functional lung imaging

To assess lung function parameters using PREFUL-MRI, we used a dedicated research software (MR Lung version 2.2, Siemens Healthineers) after automatically registering the mid-expiration position and segmenting the lung parenchyma.^35^ Normalized perfusion (expressed as a percentage), referencing a full-blood signal region, identified as the region with the highest perfusion signal between the lungs, typically reflecting the aorta or other major vessels^25^; regional ventilation (also expressed as a percentage), calculated based on the signal values at end-inspiration (insp), end-expiration (exp), and middle position (mid): $\frac{S\;\text{mid}}{S\;\text{insp}}-\frac{S\;\text{mid}}{S\;\exp}$^23^; and flow-volume loop correlation, defined as the correlation of the flow-volume loop (derived from the reconstructed ventilation cycle) relative to a healthy region (the largest connected region within the 80th and 90th ventilation percentiles).^60^ Based on these maps, the percentage of defect areas (QDP, VDP) was calculated using thresholds optimized on a large sample, all scanned at 1.5 T with a fast low-angle shot sequence. Thresholds included: perfusion (2%), fractional ventilation (40% of the 90th percentile), and flow-volume loop (0.9%). The percentage of concurrent defect areas in perfusion and ventilation (V/Q defect and V/Q defect of the flow-volume loop), as well as exclusive perfusion defects (without concurrent ventilation defects) and vice versa (exclusive ventilation defects with no concurrent perfusion defect, based on normalized perfusion and flow-volume loop correlation), were calculated. Additionally, areas without defects in both perfusion and ventilation maps (V/Q match and V/Q match based on flow-volume loop) were determined.^25^

### Quantification and statistical analysis

We used the Shapiro-Wilk test to test for normal distribution. If data was normally distributed unpaired *t* test was performed for normally distributed data. If data was not normally distributed, we performed the Mann-Whitney U test. Continuous variables are given as means with SEMs and categorical variables as numbers with percentages. The occurrence of MRI changes is given as a percentage of the study sample. Group comparisons involving more than two groups were analyzed using one-way ANOVA. Correlations are given as Spearmen (rs). Adjusted *p* values are reported. All analyses were performed using GraphPad Prism (Version 10.2.0, GraphPad Software, La Jolla, CA, USA). *p* < 0.05 was considered to indicate statistically significant difference in all analyses. In figures, ∗ indicates *p* < 0.05, and ns indicates not significant.

### Additional resources

Clinical trial registration at ClinicalTrials.gov (ID: NCT05083806), providing detailed information on the study objectives, design, and methodology: https://clinicaltrials.gov/study/NCT05083806?cond=Pompe%20Disease&intr=MSOT&rank=1.
