# Supplementary material for: Mapping lung function in late-onset Pompe disease using label-free functional MRI
Source: iScience. 2026 Feb 25;29(3):115136. doi: 10.1016/j.isci.2026.115136 (PMC13010127; doi:10.1016/j.isci.2026.115136)
Supplement: Document S1. Table S1 [file mmc1.pdf]

iScience, Volume *29*

Supplemental information

Mapping lung function in late-onset Pompe disease using label-free functional MRI

Lina Tan, Alexandra L. Wagner, Rafael Heiss, Nadine Bayerl, Jana Zschüntzsch, Adrian P. Regensburger, Frauke Alves, Matthias Türk, Sandy Schmidt, Robert Grimm, Matthias Vorgerd, Lara Schlaffke, Hannah Vogt-Wolz, Merle Claßen, Benjamin Stoecklein, Adrian Buehler, Joachim Woelfle, Michael Uder, Andreas Hahn, Alexander Mensch, Martin Winterholler, Regina Trollmann, Roman Raming, and Ferdinand Knieling

Patient ID	Start of examination (hh:mm:ss)	End of the examination (hh:mm:ss)	Total PREFUL examination time
1	12:07:32	12:09:02	1min. 30sec.
2	13:20:22	13:21:52	1min. 30sec.
3	11:51:23	11:52:53	1min. 30sec.
4	10:08:10	10:09:40	1min. 30sec.
5	11:32:59	11:34:29	1min. 30sec.
6	12:24:34	12:26:04	1min. 30sec.
7	11:33:30	11:35:02	1min. 32sec.
8	13:55:30	13:57:00	1min. 30sec.
9	11:56:00	11:57:30	1min. 30sec.
10	09:33:03	09:34:33	1min. 30sec.
11	14:49:09	14:50:39	1min. 30sec.
12	09:34:35	09:36:05	1min. 30sec.
13	14:37:17	14:38:47	1min. 30sec.
14	09:27:30	09:29:00	1min. 30sec.
15	11:32:13	11:33:43	1min. 30sec.
16	13:09:01	13:10:31	1min. 30sec.
17	14:05:51	14:07:21	1min. 30sec.
18	14:12:51	14:14:21	1min. 30sec.
19	14:36:29	14:37:59	1min. 30sec.
20	14:38:39	14:40:09	1min. 30sec.

Table S1. Overview of PREFUL MRI scanning duration, Related to STAR Methods.

For every Pompe patient, the start and end times of the examination are reported in hour (hh): minute (mm): second (ss) format. The right column shows the total PREFUL examination time, calculated as the duration between start and end times.

hat formatiert: Ausgeblendet

Formatiert: Zeilenabstand: einfach

hat formatiert: Schriftart: Englisch (Vereinigte Staaten)
